# CYP2D6 phenotype explains reported yohimbine concentrations in four severe acute intoxications

**DOI:** 10.1007/s00204-021-03082-4

**Published:** 2021-05-24

**Authors:** Anna Mueller-Schoell, Robin Michelet, Ferdinand Weinelt, Charlotte Kloft, Gerd Mikus

**Affiliations:** 1 Department of Clinical Pharmacy and Biochemistry, Institute of Pharmacy, Freie Universitaet Berlin, Kelchstr. 31, 12169 Berlin, Germany; 2 Graduate Research Training Program PharMetrX, Berlin/Potsdam, Germany; 3 Department of Clinical Pharmacology and Pharmacoepidemiology, University Hospital Heidelberg, Im Neuenheimer Feld 410, 69120 Heidelberg, Germany

**Keywords:** Yohimbine, CYP2D6, Pharmacokinetics, Clearance, Modelling & simulation, Pharmacometrics

## Abstract

The indole alkaloid yohimbine is an alpha-2 receptor antagonist used for its sympathomimetic effects. Several cases of yohimbine intoxication have been reported and the most recent one involved four individuals taking a yohimbine-containing drug powder. All individuals developed severe intoxication symptoms and were admitted to the hospital. Even though all individuals were assumed to have taken the same dose of the drug powder, toxicology analyses revealed yohimbine blood concentrations of 249–5631 ng/mL, amounting to a 22-fold difference. The reason for this high variability remained to be elucidated. We used recently reported knowledge on the metabolism of yohimbine together with state-of-the art nonlinear mixed-effects modelling and simulation and show that a patient’s cytochrome P450 2D6 (CYP2D6) phenotype can explain the large differences observed in the measured concentration after intake of the same yohimbine dose. Our findings can be used both for the identification of safe doses in therapeutic use of yohimbine and for an explanation of individual cases of overdosing.

Yohimbine is a plant-derived indole alkaloid and alpha-2 receptor antagonist (Swann et al. 2013), historically used for erectile dysfunction (Wishart et al. 2018) but also abused in the bodybuilding community (Drevin et al. 2020). Recently, it has received increased attention as a promising compound for the microdose-based identification of a patient’s cytochrome P450 (CYP) metaboliser status (Vay et al. 2020). Over the years, several cases of yohimbine intoxication have been reported (Giampreti et al. 2009; Anderson et al. 2013; Gicquel et al. 2016; Drevin et al. 2020). The most recent case report on four individuals intoxicated with yohimbine described four individuals with severe intoxication symptoms leading to hospitalisation approximately 10 h after taking a drug powder containing yohimbine (Zhu et al. 2021). Of the four individuals, three recovered after treatment and one died before arrival at the hospital. The three recovered individuals took 4–5 g of the drug powder. While no amount could be reported for the deceased person, it is likely, and we assume that the same amount was taken. From every person, a blood sample was obtained between 10 and 11 h after yohimbine intake. The concentrations measured via LC–MS/MS were 459, 249, 301, and 5631 ng/mL with the highest concentration measured in the deceased person. The authors highlighted the potential risk for yohimbine overdosing and we appreciate their important contribution to defining a therapeutic window for yohimbine. However, while the authors focused on the high interindividual variability in bioavailability as a possible explanation for the very different concentrations measured between 10 and 11 h after intake of yohimbine, we think that newly emerged knowledge on its pharmacokinetics and metabolism (Vay et al. 2020) together with pharmacokinetic simulations can further elucidate reasons for the observed high yohimbine concentrations.

There are only a few publications on the pharmacokinetics of yohimbine, whose bioavailability is highly variable (Guthrie et al. 1990) and elimination has been poorly characterised for a long time. Recently published new data identified the highly polymorphic CYP2D6 as a major contributor to the clearance of yohimbine (Vay et al. 2020). In this work, the pharmacokinetics of a single oral dose of 5 mg yohimbine was characterised in CYP2D6 genotyped Caucasian volunteers. In line with the large differences in CYP2D6 metabolic activity, the apparent oral clearance varied substantially by more than 600-fold (25 – 15,863 mL/min) and terminal elimination half-life ranged from 0.5 to 7.6 h. It is known that the CYP2D6 activity distribution is different between Caucasian and Chinese individuals. While East Asians show a lower frequency of individuals with no enzymatic activity, the frequency of decreased enzyme activity is much higher compared to Caucasians (Bertilsson et al. 1992; Huddart et al. 2019; Nofziger et al. 2020, Mueller-Schoell et al. 2021). In combination with the high dose, this reduced metabolic activity could have resulted in the highly toxic yohimbine concentrations in the four Chinese individuals.

To explore this hypothesis, we used the pharmacokinetic data published (Vay et al. 2020) to develop a nonlinear mixed-effects pharmacokinetic model of yohimbine. In short, a two-compartmental model with first-order absorption and linear elimination was fitted to the data reported by Vay et al. using the software NONMEM v. 7.4 (ICON Development Solutions, Ellicott City, MD, USA). The interindividual variability on the yohimbine clearance was largely explained by CYP2D6 activity, leading to a reduction from 1143 to 43.9 CV% after inclusion of genotype-derived phenotype as a covariate. The individual yohimbine clearance was then estimated using maximum-a-posteriori likelihood (MAP) estimation with the developed yohimbine model as prior. Subsequently, the individual model was simulated using mrgsolve (v.0.10.1) in R/Rstudio (v. 3.6.3/1.3.959). Given the information known about the four intoxicated patients (i.e., approximate amount of drug taken, approximate time of blood sampling, and measured yohimbine concentrations), the developed model was then used to estimate each of the intoxicated patients’ yohimbine clearance and most likely CYP2D6 phenotype, considering the yohimbine clearances observed in patients of different phenotypes in the study by Vay et al. For the four intoxicated patients, the time points of blood sampling were set to 10.5 h since only an approximate value was given. Furthermore, the intake of 5 mg of yohimbine was assumed for all individuals.

The measured and model-predicted concentrations at approximately 10.5 h after intake together with the predicted apparent clearances and half-lives are reported in Table 1 and the simulated yohimbine concentration–time profiles along with the measured concentrations are shown in Fig. 1.

**Table 1:** Pharmacokinetic simulation results for yohimbine in the four intoxication cases, assuming a 5 g dose and a sampling time of 10.5 h

	Case 1	Case 2	Case 3	Case 4
Measured C_10.5 h_ [ng/mL]	459	249	301	5631
*Simulation*				
CL/F [mL/min]	822.3	1013.2	927.3	403.1
t_1/2_ [h]	0.74	0.64	0.69	1.80
Predicted C_10.5 h_ Median (90%PI) [ng/mL]	349.5 (226.8–648.2)	196.5 (121.9–430.8)	240.4 (147.0–466.8)	3675.0 (2478.1–5418.5)
Median predicted C_max_ [ng/mL]	34,148	30,328	30,160	41,299

*C*_*10.5 h*_ concentration at 10.5 h after intake; *CL/F* apparent clearance; *t*_*1/2*_ half-life; *PI* prediction interval; *C*_*max*_ maximum concentration

**Fig. 1:**
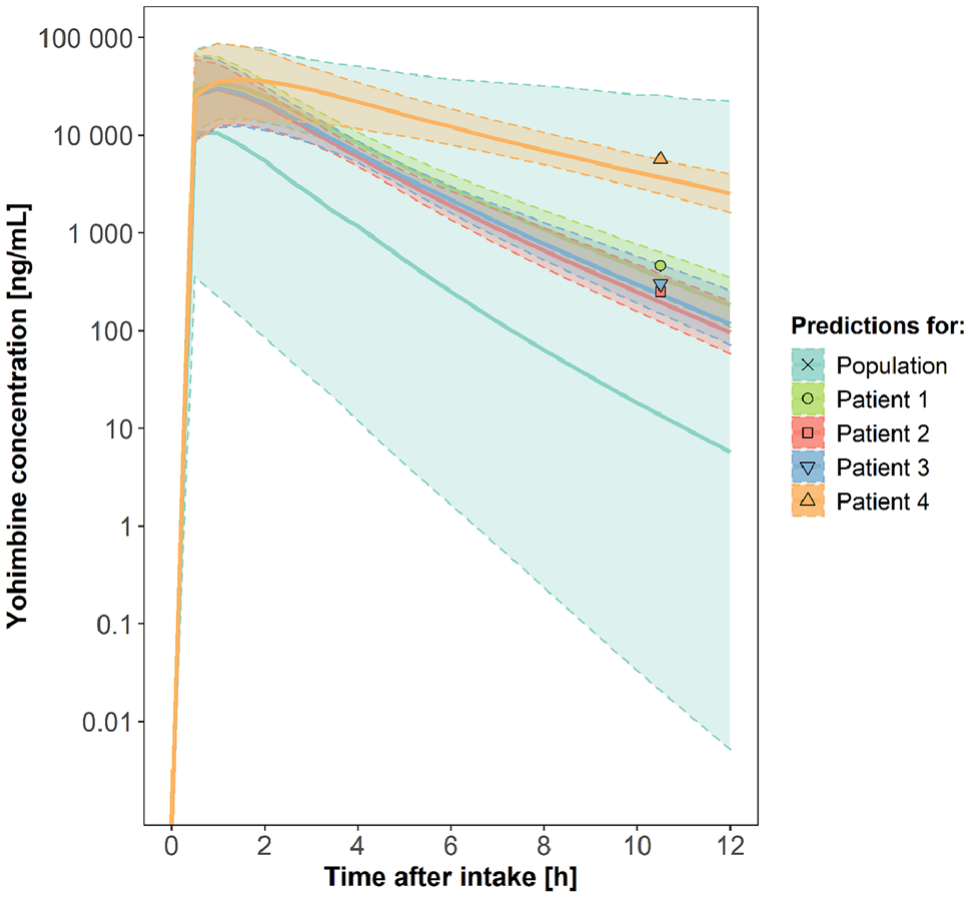
Model-predicted and observed concentrations after a single administration of 5 g yohimbine. Model predictions based on stochastic simulations (*n* = 1000) of the prior (population) or posterior (individual patient’s) variability distribution of the yohimbine pharmacokinetic model. *Solid lines:* median predicted concentrations, *dotted lines and coloured shades:* 90% prediction intervals

According to the model predictions, extensive metabolisers exhibit a yohimbine CL/F greater than 3000 mL/min whereas poor metabolisers exhibit a yohimbine CL/F below 100 mL/min. Based on the estimated clearance values, all four patients were phenotypic CYP2D6 intermediate metabolisers with decreased metabolic activity. In addition, there might have been some degree of auto-inhibition which has been reported for yohimbine before (Vay et al. 2020). Yet, since very high doses such as 5 g have never been investigated before, the degree of autoinhibition and its contribution to the observed decreased clearance is unknown and needs further evaluation.

There are several limitations associated with our study: first, while we assumed that all patients ingested 5 g of yohimbine, the precise amount of yohimbine in the drug powder was not known. The assumed dose of 5 g yohimbine supposes a purity of 100% in the drug powder, thus, it is possible that a lower amount was ingested which, given the measured concentrations, would result in even lower estimated yohimbine clearances. The dose ingested by patient four is unknown. Considering that this patient died after drug powder intake and he had a more than tenfold higher concentration compared to the other three patients, either the ingested dose was much higher or he had a lower CYP2D6 activity or a combination of both. Second, as the CYP2D6 genotypes of the four patients were not determined, we were not able to test the validity of our model predictions. While the ability of the model to well capture the observed concentrations supports its performance, the feasibility of estimating an individual’s CYP2D6 phenotype based on measured yohimbine concentrations should be confirmed with an independent dataset incorporating measured yohimbine concentrations and the CYP2D6 genotype and phenotype in the future.

In conclusion, the CYP2D6 metabolic activity plays a key role in the metabolism of yohimbine and especially individuals with decreased activity are at risk for overdosing/toxic concentrations. The usual therapeutic dose of 15–30 mg is sometimes exceeded and warnings about potentially dangerous side effects (including death) are stated without data supporting it (WebMD). Because the reported amounts taken in the case report were certainly excessively high, the outcome was not preventable. Yet, if yohimbine is used therapeutically, the consideration of an individual’s CYP2D6 phenotype/metabolic activity will reduce the risk for toxic concentrations and increase drug safety. Determining a patient’s CYP2D6 activity by phenotyping before treatment initiation can minimise the risk for individual overdosing in the therapeutic setting. Leveraging prior pharmacokinetic knowledge with forensic (or toxicology) drug monitoring in a modelling and simulation framework seems an appealing approach in elucidating individual overdosing if a CYP2D6 activity measurement is not available.

## Data Availability

Not applicable.

## Code availability

The NONMEM model code for the nonlinear mixed-effects model is available on reasonable request from the corresponding author.
